# O-linked mucin-type glycosylation in breast cancer

**DOI:** 10.1042/BST20170483

**Published:** 2018-06-14

**Authors:** Joy M. Burchell, Richard Beatson, Rosalind Graham, Joyce Taylor-Papadimitriou, Virginia Tajadura-Ortega

**Affiliations:** Breast Cancer Biology, School of Cancer and Pharmaceutical Sciences, King's College London, Innovation Hub, Guy's Hospital London, London, U.K.

**Keywords:** breast cancer, glycosyltransferases, immunotherapy, lectins, O-linked mucin-type glycosylation

## Abstract

Changes in mucin-type O-linked glycosylation are seen in over 90% of breast cancers where increased sialylation is often observed and a change from branched glycans to linear glycans is often seen. There are many mechanisms involved including increased/altered expression of glycosyltransferases and relocalisation to the endoplasmic reticulum of the enzymes responsible for the addition of the first sugar, *N*-acetyl-d-galactosamine. It is now becoming clear that these changes can contribute to tumour growth and progression by modulating the micro-environment through glycan-sensing lectins expressed on immune cells, by modulating interactions with tumour surface receptors and by binding to selectins. The understanding of how changes in mucin-type O-linked glycosylation influence tumour growth and progression reveals new potential targets for therapeutic intervention in the treatment of breast cancer.

## Introduction

While genomics and proteomics are producing unparalleled discoveries that are advancing the understanding of biological processes and cancer, this is often incomplete without a knowledge and understanding of post-translational modifications (PTMs) of proteins, which greatly increase the size, diversity and function of the proteome [1]. Glycosylation is the most abundant PTM and in eukaryotes the majority of proteins that are expressed on the cell membrane or are secreted carry glycans, one of the four fundamental macromolecular components of all cells, together with nucleic acid, proteins and lipids. Glycoproteins carry glycans covalently attached to the peptide backbone, usually via nitrogen (N-linked) or oxygen (O-linked) linkages [2].

N-linked glycans are attached to asparagine within the consensus sequon Asn-X-Ser/Thr, where X is any amino acid except proline. The linkage sugar in eukaryote *N*-glycans is *N*-acetyl-d-glucosamine (GlcNAc) and the sugars are added en bloc in the endoplasmic reticulum. During trafficking through the secretory pathway sugars can be trimmed and added to give the final glycan side chain [3].

In contrast with *N*-glycans, *O*-linked glycans are added to the peptide core individually and sequentially [4]. There are many different types of O-linked glycosylation in which different sugars are O-linked to serine or threonine, for example *O*-fucose, *O*-mannose, *O*-glucose, *O*-xylose [2]. Moreover, GlcNAc can be O-linked to serine or threonine on proteins found in the nucleus, cytoplasm and mitochondria [5]. However, this review will be confined to mucin-type O-linked glycosylation, where *N*-acetyl-d-galactosamine (GalNAc) is added in O-linkage to serine or threonine, and the changes that occur in this type of glycosylation in breast cancer. For a more extensive review on mucin-type glycosylation in cancer in general please see the reviews by Kudelka et al. [6] and Pinho and Reis [7].

Aberrant glycosylation occurs in essentially all types of human cancer and glycosyltransferase (GT) gene expression can be used to classify cancer subtypes [8]. Although aberrant glycosylation occurring in tumours has been recognised for over forty years [9], it is relatively recent that its critical role in tumour growth and progression has been recognised. Indeed glycan changes contribute to the malignant phenotype, metastasis and immune evasion (see below). Changes in O-linked mucin-type glycosylation are observed in over 90% of breast cancers, as demonstrated, for example, by the expression of the Tn antigen [10] and the loss of core 2 glycans [11]; see below. Such selectivity throughout cancer evolution suggests that mucin-type O-linked glycosylation may be a driver of tumour progression.

## Mucin-type O-linked glycosylation

Mucin-type O-linked glycosylation is so-called as it was originally found abundantly on mucins, which carry many and often hundreds of glycans in this linkage [4]. However, this type of glycosylation is also found on many other types of cell membrane glycoproteins where only 1 or 2 sites may be present [12].

**Figure 1.**
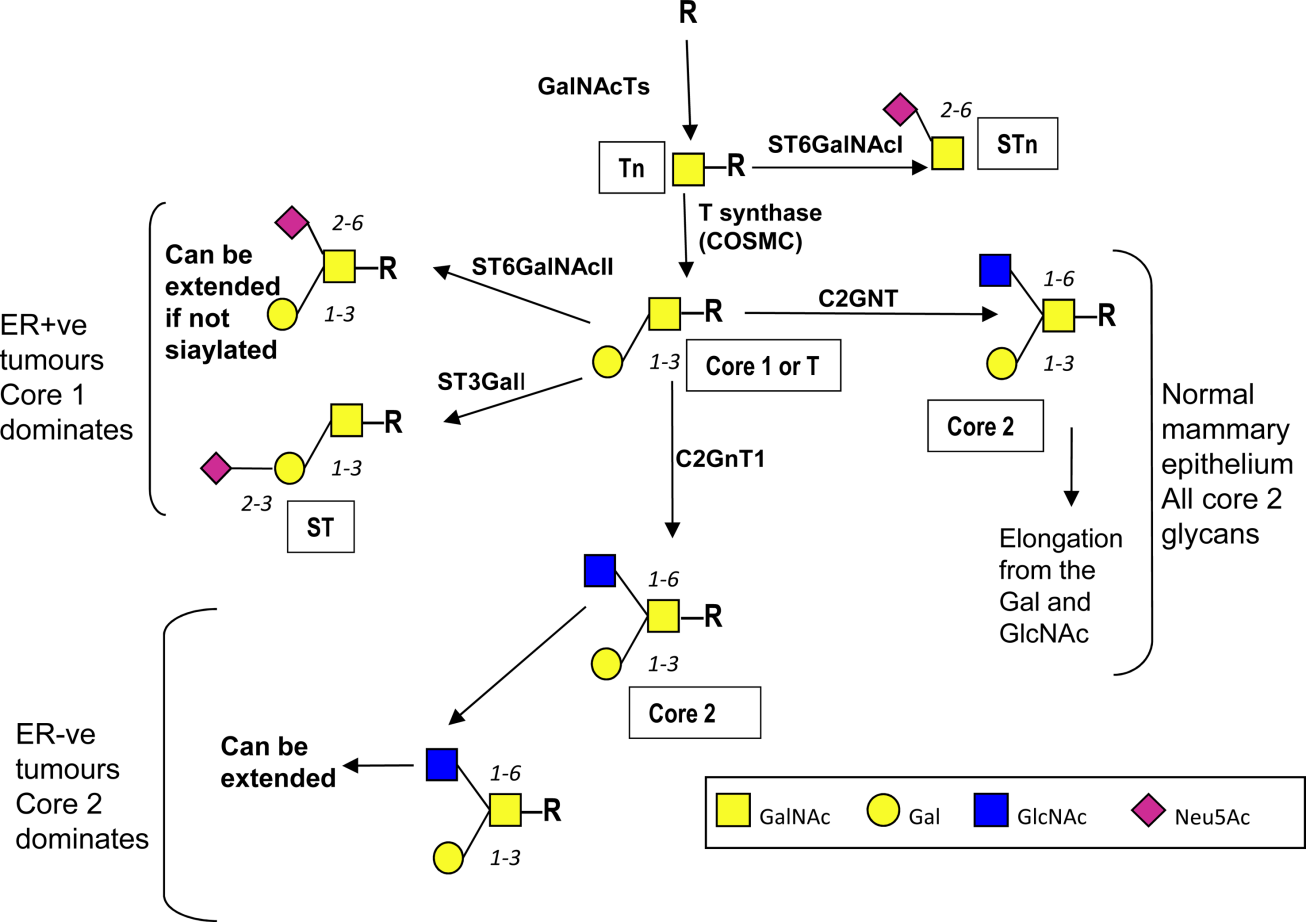
Pathways of O-GaalNAc glycosylation in the normal and malignant mammary gland. In the normal mammary gland epithelial cells O-GalNAc glycans are core 2-based and extension can occur from the Gal and the GlcNAc. COSMC is indicated under the T synthase as it acts as a private chaperone for this enzyme and hence is required for T synthase activity ([21], see text). In many breast cancers truncated mucin-type O-linked glycans are seen often terminating in sialic acid due to up-regulation of sialyltransferases [16]. However, in ER-ve breast cancer core 2-based glycans are expected to be present and may dominate due to the overexpression of the *C1GALT1* and *GCNT1* in ER-ve tumours compared with ER+ve breast cancers [20]. Symbols used are based on the nomenclature recommended in Varki et al. [79].

Mucin-type O-linked glycosylation (for clarity and brevity from now on referred to as O-GalNAc glycosylation) is characterised by the addition of GalNAc to serine and threonine, which is mediated by a large family of polypeptide *N*-acetylgalactosaminyltransferases (GalNAc-Ts), and, in contrast with N-linked glycosylation and most other types of O-linked glycosylation, is initiated in the Golgi apparatus [13]. After the addition of the first sugar many so-called core structures can be formed. In mammary gland epithelial cells the addition of galactose (Gal) results in the formation of core 1 which is converted into core 2 by the addition of GlcNAc to form the branched core 2 structure ([4,14]; see Figure 1). Extension of the glycan chains can then occur from the GlcNAc and/or the Gal; see Figure 1.

## Changes in O-GalNAc glycosylation seen in breast cancer

In breast cancer there can be a change in the number of O-GalNAc glycans added to the peptide core of glycoproteins, as well as changes in the core structures, which often results in increased sialylation [7,15–17]. Overall there is change from core 2-based glycans to more simple glycans with many breast cancers showing exposure of the first sugar GalNAc (Tn) and the disaccharide which forms core 1 (T), as originally described by Springer in 1984 [18]. The sialylated derivatives of these glycans are also commonly observed, giving the sialylated Tn (STn) and sialylated core 1 (ST) glycans; see Figure 1. The readers are referred to Ju et al. [19] for a comprehensive review of the Tn and STn antigens in cancer. However, estrogen receptor-positive (ER+ve) and estrogen receptor-negative (ER-ve) breast cancers show different profiles of genes encoding GTs associated with O-linked glycosylation [20]. These data suggest that ER+ve breast cancer, which accounts for ∼75% of all breast cancers, carry mostly simple core 1-based glycans on their O-linked glycoproteins, whereas ER-ve cancers can also carry the branched elongated core 2 glycans associated with a normal glycophenotype [17,20].

## Mechanisms responsible for aberrant O-GalNAc glycosylation

There are many mechanisms that have been shown to contribute to changes in O-GalNAc glycosylation in breast cancer including relocalisation of GalNAc-Ts, changes in expression of GTs as well as altered glycosidase activity. There is a single enzyme responsible for adding Gal to GalNAc to form the core 1 glycan (T antigen) and this is known as T synthase (core 1 β3-galactosyltransferase) encoded by the *C1GALT1* gene (see Figure 1). A private chaperone is required for the correct folding and activity of T synthase and this is known as Cosmc. Although mutations in *COSMC* and methylation of its promoter play a role in the expression of Tn in cervical and pancreatic cancers, respectively [21,22], this does not appear to be the case for breast cancers [23–25].

### Changes in expression of GTs: GalNAcTs

There are twenty GalNAcTs (GalNAcT1-T20) that are capable of initiating O-GalNAc glycosylation and each has distinct peptide substrate specificities although some overlap exists [13]. Each tissue can express a specific profile of GalNAcTs, and GalNAcT1 and GalNAcT2 (encoded by *GALNT1* and *GALNT2*), which are often thought of as ‘house-keeping' GalNAcTs, are expressed in normal mammary epithelial cells [26,27]. However, in breast cancer increased expression of other GalNAcTs is seen and these include GalNAcT6 [27] and GalNAcT14 [28]. GalNAcT6 (encoded by *GALNT6*) is expressed in the majority of breast cancers [26,29,30] but no expression was seen in sections of normal breast tissue [26,30]. In breast cancer GalNAcT6 can glycosylate and stabilise the MUC1 mucin [30], resulting in increased proliferation and decreased cell adhesion [30]. Interestingly, GalNAcT6 is also expressed in colon adenocarcinoma but not normal colon, and its expression results in dysplastic growth [31]. By immunohistochemistry, GalNAcT14 is expressed in 84% of breast cancers, while only being found in 14.6% of non-malignant breast tissue [28]. Its expression is associated with grade, but the functional significance has yet to be elucidated.

### Relocalisation of GalNAcTs results in the increased expression of Tn

**Figure 2.**
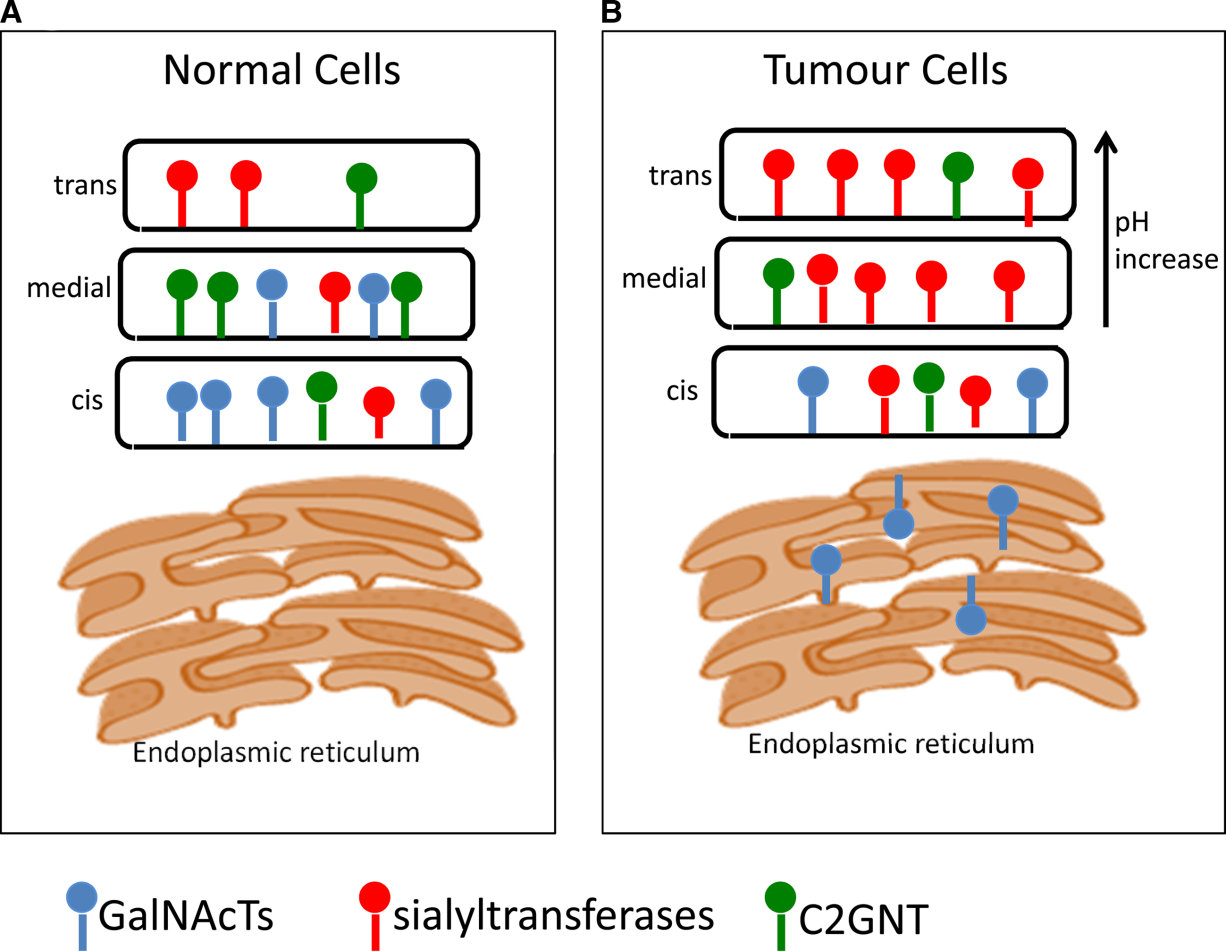
Mucin-type O-GalNAc glycosylation is initiated in the Golgi. (**A**) Normally O-GalNAc glycosylation is initiated in the Golgi. GalNAcTs are not confined to the *cis*-Golgi, and ST3GalI and C2GNT overlap to some degree [80]. (**B**) In the malignant cell some GalNAcTs can be relocated to the endoplasmic reticulum where normally unmodified serine/threonines in proteins can be modified with GalNAc by the ER-relocated GalNAcTs. This results in increasing the density of glycosylation and expression of Tn. This relocation process is termed the GALA pathway [24,32,33]. ST3GalI is overexpressed in 90% of breast cancers [16], and ST6GalNAcI is seen in 25% of breast cancers [34]. Changes in pH across the Golgi cisternae can also be observed in breast cancer [43,44]. For clarity only GalNAcTs, sialyltransferases and C2GNT are depicted.

O-GalNAc glycosylation is initiated by the GalNAcTs localised within the Golgi apparatus [13]; see Figure 2A for a simplified diagram of the Golgi apparatus. However, recent data suggest that GalNAcTs can relocate to the endoplasmic reticulum in some cancer cells and that this is driven by Src kinase regulating the COPI transport machinery [24,32]; see Figure 2. This relocation results in the increased expression of the Tn antigen [24,32]. Staining of breast cancer tissue microarrays (TMAs) with the Vicia Villosa lectin (VVA), which recognises Tn, showed an endoplasmic reticulum localisation of Tn in 70% of the cancers [24], suggesting O-GalNAc glycosylation had been initiated in this organelle. Moreover, there was a marked effect on cell motility and invasiveness with endoplasmic reticulum localised GalNAcT2 [24]. Interestingly, in an *in vivo* murine model of liver cancer, endoplasmic reticulum localisation of GalNAcT1 increased tumour growth driven by increased glycosylation of MMP14 [33].

### Changes in expression of GTs: the core forming and extension GTs

#### Expression of ST6GalNAcI to form STn

The Tn antigen can be converted into STn by the action of ST6GalNAcI. This is due to the transcriptional activation of *ST6GALNACI* in ∼25% of breast carcinomas where there is a complete concordance between mRNA expression and the glycan being seen in the cancers [34]. However, in studies using only immunohistochemistry with different antibodies to define the presence of STn in breast cancers, there is little agreement on the percentage of breast cancers carrying this glycan [35]. Nevertheless, *in vitro* and *in vivo* experiments with breast cancer cell lines indicate that STn is associated with tumour progression and migration [36], and in primary breast cancers STn expression is associated with resistance to adjuvant chemotherapy in node-positive patients [37].

#### Increased expression of ST3GalI and ST6GalNAcII to form sialylated and disialylated core 1

The core 2 branch seen on O-linked glycoproteins expressed by the normal mammary epithelial cells is initiated by the action of the GT C2GNT (encoded *by GCNT1*; see Figure 1). This enzyme uses as a substrate Galβ1,3GalNAc known as core 1 or T, which is synthesised by the action of T synthase. Galβ1,3GalNAc (core 1) also forms the substrate for the competing sialyltransferases ST3GalI and ST6GalNAcII (Figure 1), which terminate chain extension. Thus, the relative expression of these GTs determines whether glycoproteins with O-GalNAc glycans carry core 2 structures that allow further extension, or sialylated core 1 glycans [38] such as ST. Although ST3GalI expression is elevated in 90% of breast cancers [16], the ST glycan is found in ∼50% of these cancers due to competition with C2GNT, which is only lost in a proportion of breast cancers [16]. In addition, the presence of T is often seen [23]. Moreover, in breast cancer subtypes, changes are seen in both the relative levels of core 1 versus core 2 structures and in the terminal units carried by these structures. Thus, as predicted from profiles of GTs expressed, ER+ve breast cancers show a dominance of core 1-based glycans [20] and sialylated cores (ST) due to an increased expression of ST3GalI. On the other hand, in ER-ve breast cancers, core 2-based structures appear to be dominant [20]. In these cancers the core 2 structures can be carrying the sialyl Lewis X (sLe^X^) glycan, which is not found on core 2 in the normal breast. Selectin interactions with glycans including sLe^X^ are crucial to immune cell trafficking [39]. It therefore appears that breast cancers have hijacked this normal lectin/glycan interaction to aid in the metastatic process (see below).

Higher levels of expression of ST6GalNAcII, which forms disialylated core 1 (see Figure 1), are associated with ER+ve compared with ER-ve breast cancers. However, high levels of this sialyltransferase are associated with an increased survival time in ER-ve tumours [40]. This sialyltransferase has been identified as a metastasis suppressor gene as its knockdown results in an increase in metastasis in an *in vivo* model [40]. Evidence suggests that the addition of sialic acid in the α2,6-position to the core 1 structure inhibits the cells' ability to bind to galectin-3, a lectin associated with many processes involved in tumour progression and metastasis [40].

### Changes in expression of glycosidases

α-l-fucosidase 1 (FUCA1) is found in lysosomes and catalyses the removal of terminal fucose. The mRNA encoding this enzyme is highly overexpressed in breast cancer compared with normal breast tissue but is associated with early-stage tumours [41]. This association with early-stage disease of the fucosidase may be due to fucose being part of Le^X^ glycan, the sialylated version of which binds to selectins and may play a role in metastasis. Moreover, higher levels of FUCA1 predicted a better overall survival in patients with triple negative breast cancer [41], which by definition, is ER-ve.

### Changes in pH of the Golgi apparatus and availability of sugar nucleotides

Regulation of pH within organelles is essential for their correct physiological function, and the pH of the Golgi apparatus is strictly controlled [42]. The presence of the unsubstituted T antigen (core 1) in most breast cancers [23] is difficult to explain by changes in expression of GTs. However, small increases in pH within the Golgi apparatus have been associated with an increased presence of the T antigen in cell lines [43], which may be the result of mislocalisation of certain GTs within the Golgi [44]; see Figure 2.

Another possible contributing factor to aberrant mucin-type glycosylation is that there may be a change in the availability of the nucleotide sugar donors, and elevated levels of UDP-GlcNAc and UDP-GalNAc have been found in breast cancer cell lines [45].

## Role of changes in mucin-type O-GalNAc glycosylation in tumour growth and metastasis

**Figure 3.**
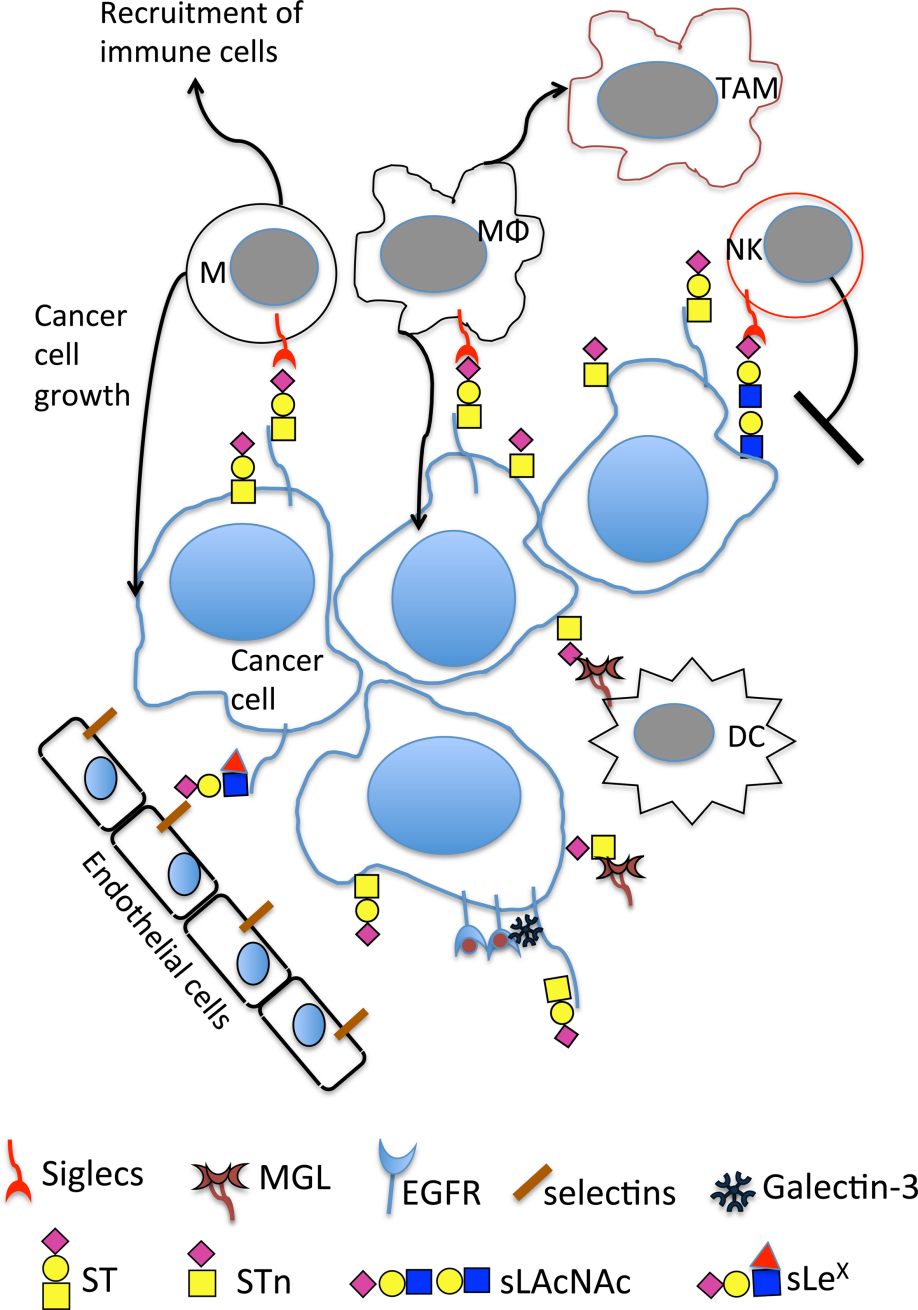
Mucin-type O-GalNAc glycosylation changes seen in breast cancer lead to increase in tumour growth and progression through a number of mechanisms. O-GalNAc changes lead to novel interactions with lectins on immune cells including the binding of sialylated glycans to siglecs on monocytes, macrophages and NK cells. This includes the binding of MUC1-ST to siglec-9 on monocytes and macrophages and the binding of sialylated LacNAc (which can be carried on core 1 and core 2 branches) to siglec-7 on NK. Binding of Tn and STn to MGL on dendritic cells and macrophages is also observed. Expression of sLe^X^ can lead to binding to selectins on endothelial cells, and different core glycans can dictate how cancer cells respond to EGF binding. M, monocytes; MΦ, macrophages; TAM, tumour-associated macrophages; NK, natural killer cells; DC, dendritic cells; MGL, macrophage galactose-specific lectin; EGFR, epidermal growth factor receptor. Symbols for the glycans are as in Figure 1 with the addition of the red triangle for fucose.

The presence of tumour-associated carbohydrate antigens is generally associated with a poor prognosis and reduced overall survival [46]. In O-GalNAc glycosylation overexpression of ST3GalI leading to the increased expression of sialylated core 1 (ST) by tumour cells leads to increased tumour growth in transplantable [47] and spontaneous models of breast cancer [48]. Moreover, loss of core 1 *O*-glycans in spontaneous mammary cancer models delayed the onset and growth of the tumours and impaired the localisation of Muc1 [49]. Data are now emerging as to the mechanisms involved in this accelerated initiation and tumour growth by core 1 and sialylated core 1 glycans; see Figure 3.

### Modification of the immune micro-environment

The dramatic changes seen in O-GalNAc glycosylation in breast cancers leads to new interactions with carbohydrate-binding lectins expressed by immune cells [50]. This allows the immune compartment to respond to changes in glycosylation, which often leads to activating inhibitory pathways.

As described above, increased sialylation in breast and many other cancers is a common change seen in malignancy. Importantly, both the Tn and STn glycans can bind to the macrophage galactose-specific lectin (MGL) expressed by macrophages and dendritic cells [23], inducing inhibitory signals resulting in effector T-cell apoptosis [51].

Although Tn is highly expressed by most breast cancers, it is mainly observed inside the cells [23,24]. STn, on the other hand, is found at the cell surface in ∼25% of breast cancers and hence can interact with MGL on dendritic cells and macrophages [23,34].

Sialic acid patterns on the cell surface can be recognised as ‘self' by the immune system, and the term SAMPs (self-associated molecular patterns) has been coined to describe these glycans [52]. Siglecs (sialic acid-binding immunoglobulin-like lectins) are a family of sialic acid-binding lectins mainly expressed on cells of the immune system that have evolved to recognise these SAMPs and deliver signals that negatively regulate the immune system [53]. Like the immune checkpoint receptor PD1 (programmed cell death 1), many siglecs contain ITIMs (immunoreceptor tyrosine-based inhibition motif) within their cytoplasmic tails [53]. It has recently become apparent that siglecs play a role in cancer immune suppression, the hypersialylation seen in cancers inducing binding to these lectins [54–58].

Siglec-7 is expressed by NK cells, and increased sialylation on tumour cells inhibits NK activation through binding to this siglec [56]. Interestingly, the use of trastuzumab, an antibody to HER-2 used in the clinic for the treatment of HER-2 positive breast cancers, to deliver sialidase to tumour cells reduced binding to siglec-7 and enhanced NK killing [57].

MUC1 is a mucin that is highly glycosylated with *O*-GalNAc glycans and is overexpressed and aberrantly glycosylated in greater than 90% of breast cancers [11]. A tumour-associated glycoform of MUC1, MUC1-ST can interact with siglec-9 expressed by monocytes and macrophages, resulting in the secretion of many factors that are involved in tumour progression and immune recruitment [58]. The binding of MUC1-ST to siglec-9 on macrophages results in the expression of a tumour-associated macrophage (TAM) type phenotype [58], which is associated with poor prognosis in breast cancer [59]. These TAMS show increased expression of the programmed death ligand 1 that binds to the immune checkpoint receptor PD1 expressed by T cells. Antibody blocking of this immune checkpoint is now in clinical use with some extremely encouraging results particularly in melanoma and lung cancer [60]. The binding of siglec-9 to MUC1 has also been reported to increase the growth of the tumour cell via recruitment of β-catenin [61]. Thus the siglec glyco-immune checkpoint is a prime target for the development of novel immunotherapies [50,62,63].

### Role of aberrant mucin O-GalNAc glycosylation in tumour growth and progression of breast cancer

Glycoproteins that carry large amounts of *O*-GalNAc glycans, such as MUC1 are often overexpressed in cancer and contribute to the bulk of the glycocalyx facilitating integrin clustering that enhances interactions with ligands [64]. Thus, a bulky glycocalyx may contribute to tumour growth by enhancing integrin-mediated cell growth and survival [64].

Galectins are a family of carbohydrate-binding proteins that display a plethora of functions. Galectin-3 is elevated in the sera of breast and other cancer patients [65] and it has been shown to interact with MUC1 carrying the core 1 glycan (T glycan), enhancing tumour cell aggregation [66,67] and promoting adhesion to endothelial cells by inducing polarisation of MUC1, and thus exposing adhesion molecules [66]. EGF binding to EGFR expressed by breast cancer cells carrying core1- or core2-based glycans induces different patterns of gene expression (Tajadura-Ortega et al. submitted for publication). The mechanism responsible involves a MUC1/EGFR complex formation with galectin-3. Moreover, it has recently been shown that MUC1–galectin-3 interaction enhances the MUC1 interaction with EGFR, leading to increased dimerisation of EGFR and subsequent signalling [68].

The glycan sLe^X^ can be carried on core 1 and core 2 branches, as well as N-linked glycans. In breast cancers, its expression has been associated with a high risk of metastasis [46], although others have found no association with disease survival [20]. The combination of sLe^X^ and one of its carrier glycoproteins, (BST-2) is associated with a higher risk of brain and liver metastasis, and a 3-fold decrease in disease survival in ER-ve breast cancer patients [69]. However, although sLe^X^ is found on a higher number of ER-ve breast cancers, when high levels are expressed by ER+ve breast cancers, there is an association with metastasis to the bone [20]. Importantly, bone microvasculature cells are known to express E-selectin, one of the receptors for sLe^X^ [70], and selectin binding blocking strategies are now actively being explored as a therapeutic option in cancer [71]. These data indicate that it is the context in which sLe^X^ is found rather than the glycan itself that dictates metastatic tropism.

## Summary

Changes in O-linked glycosylation are a common feature of many cancers, including breast cancer, and result from many mechanisms. There is now convincing evidence that this is not just a passenger effect but can contribute to driving the malignant phenotype and cancer progression ([72] and see above). Understanding the mechanisms involved has allowed the development of novel therapeutics such as blocking glyco-immune checkpoints as described above. In addition, other types of immunotherapy such as the development of chimeric antigen receptors (CARs) specific for tumour-associated *O*-GalNAc glycoforms are being developed [73,74]; see reviews by Taylor-Papadimitriou et al. [75] and Steentoft et al. [76].

As in many fields, it is becoming clear that a reductionist approach will not adequately explain the complex interactions between glycans and glycan-binding proteins, glycan:glycan interactions and cell:cell interactions mediated by glycans. Within the breast cancer micro-environment cross-talk occurs between cancer cells, immune cells and stromal cells [77], often mediated by glycan-binding proteins [72]; therefore complex co-culture methods will be needed to really investigate and dissect the role of aberrant mucin-type glycosylation in breast cancer. Moreover, the recent developments in mass spectrometry allowing the collection of molecular information including glycans from formalin-fixed paraffin-embedded tissue and displaying the data as intensity maps should prove a great technological advance to the analysis of the glycome [78]. As our understanding of how changes in glycosylation can affect tumour growth and progression increases, the potential to develop further therapeutic approaches for the treatment of breast cancer will increase.

## Abbreviations

CARs: chimeric antigen receptors
ER: estrogen receptor
FUCA1: α-l-fucosidase 1
GalNAc: *N*-acetyl-d-galactosamine
GalNAc-Ts: *N*-acetylgalactosaminyltransferases
GlcNAc: *N*-acetyl-d-glucosamine
GTs: glycosyltransferases
MGL: macrophage galactose-specific lectin
NK: natural killer cells
PD1: programmed cell death 1
PTMs: post-translational modifications
SAMPs: self-associated molecular patterns
sLe^X^: sialyl Lewis X
STn: sialylated Tn
TAM: tumour-associated macrophage
